# Development of a Method for Simultaneous Generation of Multiple Genetic Modification in *Salmonella enterica* Serovar Typhimurium

**DOI:** 10.3389/fgene.2020.563491

**Published:** 2020-09-24

**Authors:** Wenxian Jing, Juan Liu, Shanshan Wu, Qiwei Chen, Xuerui Li, Yongsheng Liu

**Affiliations:** State Key Laboratory of Veterinary Etiological Biology, Lanzhou Veterinary Research Institute, Chinese Academy of Agricultural Sciences, Lanzhou, China

**Keywords:** gene modification, simultaneous construction, seamless assembly system, red homologous recombination, *Salmonella enterica* serovar Typhimurium

## Abstract

To comprehensively analyze bacterial gene function, it is important to simultaneously generate multiple genetic modifications within the target gene. However, current genetic engineering approaches, which mainly use suicide vector- or λ red homologous recombination-based systems, are tedious and technically difficult to perform. Here, we developed a flexible and easy method to simultaneously construct multiple modifications at the same locus on the *Salmonella enterica* serovar Typhimurium chromosome. The method combines an efficient seamless assembly system *in vitro*, red homologous recombination *in vivo*, and counterselection marker *sacB*. To test this method, with the seamless assembly system, various modification fragments for target genes *cpxR*, *cpxA*, and *acrB* were rapidly and efficiently constructed *in vitro*. *sacBKan* cassettes generated via polymerase chain reaction were inserted into the target loci in the genome of *Salmonella* Typhimurium strain CVCC541. The resulting pKD46-containing kanamycin-resistant recombinants were selected and used as intermediate strains. Multiple target gene modifications were then carried out simultaneously via allelic exchange using various homologous recombinogenic DNA fragments to replace the *sacBKan* cassettes in the chromosomes of the intermediate strains. Using this method, we successfully carried out site-directed mutagenesis, seamless deletion, and 3 × FLAG tagging of the target genes. This method can be used in any bacterial species that supports *sacB* gene activity and λ red-mediated recombination, allowing in-depth functional analysis of bacterial genes.

## Introduction

*Salmonella enterica* serovar Typhimurium is a foodborne and waterborne pathogen that causes gastrointestinal diseases in both humans and animals (Fabrega and Vila, 2013; LaRock et al., 2015). With the ready availability of bacterial high-throughput sequencing data generated via transcriptomic and genomic analyses, an increasing number of genes that play important roles in the antibiotic resistance and pathogenicity of *Salmonella* Typhimurium are being discovered, all of which must be functionally analyzed (Fricke et al., 2011; Leekitcharoenphon et al., 2016; Li et al., 2017). In-depth analysis of gene function requires efficient methods to simultaneously construct multiple genetic modifications, including site-directed mutation, seamless gene deletion, and insertion of exogenous sequences.

The classic λ red homologous recombination system is a simple and effective technique for gene deletion based on the λ red bacteriophage (Datsenko and Wanner, 2000; Murphy, 2016). In bacterial cells expressing λ red recombinase enzymes, a polymerase chain reaction (PCR)-amplified fragment containing a selectable antibiotic resistance gene flanked by short (∼40 bp) sequences with homology to the target region can be used to efficiently replace a gene by homologous recombination (Datsenko and Wanner, 2000; Yu et al., 2000). For markerless gene deletion, the integrated antibiotic resistance cassette can generally be removed using the FLP recombinase system (Datsenko and Wanner, 2000). However, an 82–85 bp scar sequence remains in the chromosome after the antibiotic resistance cassette is deleted, making it difficult to construct polygenic deletion mutants and excluding the prospect of seamless gene editing (Datsenko and Wanner, 2000).

Gene modification methods based on counterselection markers (such as the suicide vector strategy) make it possible to generate genetic modifications without leaving any exogenous DNA. These scarless and markerless genetic modification methods generally include two rounds of recombination involving integration and then loss of the counterselection marker (Schafer et al., 1994; Kang et al., 2002a). Common counterselection markers include levansucrase gene *sacB* (Steinmetz et al., 1985), thymidylate synthase gene *thyA* (Wong et al., 2005; Stringer et al., 2012), tetracycline repressor protein gene *tetA* (Li et al., 2013), and galactokinase gene *galK* (Warming et al., 2005). Although these methods effectively generate genetic modifications, the recombination events require the presence of long homologous arms to ensure sufficient recombination efficiency. In addition, the second recombination will either restore the wild-type allele or fix the mutant allele in the bacterial chromosome (Hmelo et al., 2015), and some counterselection markers, including *thyA*, *tetA*, and *galK*, can only be used in host strains with a particular genetic background (Warming et al., 2005; Stringer et al., 2012; Akiyama et al., 2013).

Several more effective methods that couple the λ red homologous recombination system with a counterselection marker (Gerlach et al., 2009; Stringer et al., 2012), the I-*Sce*I endonuclease (Yang et al., 2014) or the clustered regularly interspaced short palindromic repeats (CRISPR)/CRISPR-associated protein 9 (Cas9) system (Pyne et al., 2015) have also been developed. Chromosomal modifications, including scarless deletion, site-directed mutation, and epitope tagging of genes, have successfully been achieved via these methods. However, these approaches have several limitations for the simultaneous construction of multiple genetic modifications in *Salmonella* Typhimurium: (i) traditional cloning methods, such as short synthetic DNA fragment or overlap extension PCR, are inefficient, and generating multiple modifications can be time-consuming (Gerlach et al., 2009; Stringer et al., 2012) and (ii) using two or more helper vectors makes it difficult to successfully cure the plasmids from the cells (Yang et al., 2014; Pyne et al., 2015).

The recent introduction of seamless assembly methods has increased the efficiency of constructing vector-based gene modification constructs. Seamless methods are restriction and ligation independent and overcomes some of the drawbacks of traditional approaches, including the inability to assemble multiple fragments and the presence of residual scars or other short sequences (Moradpour and Abdulah, 2017). Seamless methods can be used to efficiently and rapidly assemble different DNA fragments into a plasmid and seamlessly construct synthetic and native DNA fragments (Gibson, 2011; Moradpour and Abdulah, 2017). Although gene function analysis can be performed using chimeric plasmids containing various gene modification fragments, the generation of strains with chromosomal modifications remains the most direct method of functional gene analysis.

In this study, we developed a more flexible and simple strategy for simultaneous generation of multiple genetic modifications at the same locus by coupling efficient *in vitro* (seamless cloning and assembly system) and *in vivo* (λ red homologous recombination) recombination systems. With the use of *sacBKan-*containing intermediate strains, various vector-based recombinogenic DNA fragments constructed via seamless cloning and assembly were used to simultaneously replace the *sacBKan* cassette using only traditional λ red-mediated homologous recombination. We simultaneously constructed stable site-directed mutations, seamless gene deletions, in-frame deletion, and epitope-tagged chromosomal genes in the *Salmonella* Typhimurium chromosome.

## Materials and Methods

### Bacterial Strains, Plasmids, and Culture Conditions

All strains and plasmids used in this study are listed in Table 1. *Salmonella* Typhimurium strain CVCC541, designated strain JS in a related report (Huang et al., 2016), was used for all genetic modification experiments. Trans1-T1 phage-resistant chemically competent *Escherichia coli* cells (TransGen Biotech, Beijing, China) were used for vector construction. Low-copy-number plasmid pSTV28 (Takara Bio, Shiga, Japan) was used as a cloning vector for all DNA manipulations. pUC57-*sacB*, containing the counterselection marker *sacB* (GenBank accession no: X02730.1), was artificially synthesized by our laboratory (GenScript, Nanjing, China).

**TABLE 1:** *Salmonella* strains and plasmids used in this study.

Strain or plasmid	Description	Reference or source
**Strains**		
JS	*S. enterica* serovar Typhimurium CVCC541	Supplied by China Institute of Veterinary Drug Control
JS*CpxR*:*sacBKan/pKD46*	JS containing *sacBKan* cassette replacing *cpxR* and the helper vector pKD46	This study
JS*CpxR_*D*51*A*_*	JS containing CpxR with site-specific mutagenesis (D51A)	This study
JS*CpxR_*M*199*A*_*	JS containing CpxR with site-specific mutagenesis (M199A)	This study
JS*CpxR_*D*51*A/M*199*A*_*	JS containing CpxR with double-site mutagenesis (D51A and M199A)	This study
JS*CxpR*_*SD*_	JS containing CpxR with seamless deletion	This study
JS*CpxR_*N–*3×*FLAG*_*	JS containing CpxR with 3 × FLAG tagging at the N-terminus	This study
JS*CpxA*:*sacBKan/pKD46*	JS containing *sacBKan* cassette replacing *cpxA* and the helper vector pKD46	This study
JS*CpxA_*L*38*F*_*	JS containing CpxA with site-specific mutagenesis (L38F)	This study
JS*CpxA*_Δ 92–104_	JS containing CpxA with seamless deletion at amino acids 92–104	This study
JS*CpxA_*C–*3×*FLAG*_*	JS containing CpxA with 3 × FLAG tagging at the C-terminus	This study
JS*acrB*:*sacBKan/pKD46*	JS containing *sacBKan* cassette replacing *acrB* and the helper vector pKD46	This study
JS*acrB_*D*408*A*_*	JS containing acrB with site-specific mutagenesis (D408A)	This study
JS*acrB*_*SD*_	JS containing acrB with seamless deletion	This study
**Plasmids**		
pKD46	Temperature sensitive red-expressing plasmid	Datsenko and Wanner, 2000
pUC57-sacB	Plasmid containing levansucrase gene *sacB* cassette	This study
pSTV28	Low-copy clone vector: Cm^*r*^	Takara
psacBKan	Derived from pSTV28 by inserting *sacB* and *Kan* gene, Cm^*r*^	This study

All strains were cultured aerobically in Luria-Bertani (LB) medium [1% (w/v) tryptone, 0.5% (w/v) yeast extract, and 1% (w/v) NaCl] supplemented with chloramphenicol (50 μg/ml), kanamycin (50 μg/ml), or ampicillin (100 μg/ml), as required. All growth medium components were purchased from Difco (Detroit, MI, United States). Recombinant strains were stored in 20% (v/v) glycerol at −80°C.

### DNA Manipulation

PrimeSTAR Max DNA polymerase (Takara Bio), with a mismatch rate of 12 bases per 542,580 total bases, was selected to minimize errors in the PCR products. Genomic DNA fragments were purified using a MiniBEST Bacteria Genomic DNA Extraction Kit (Takara Bio) and a MiniBEST Agarose Gel DNA Extraction Kit (Takara Bio), while plasmid DNA samples were purified using a MiniBEST Plasmid Purification Kit (Takara Bio). PCR assays were performed using PrimeSTAR Max DNA Polymerase (Takara Bio). Restriction and T4 DNA ligase enzymes were purchased from TransGen Biotech. The *in vitro* seamless assembly of different DNA fragments was performed using a pEASY-Uni Seamless Cloning and Assembly Kit (TransGen Biotech) according to the manufacturer’s instructions.

### Preparation of Recombinogenic DNA Fragments

All oligonucleotides used for mutant construction are listed in Supplementary Table S1. The pEASY-Uni Seamless Cloning and Assembly Kit (TransGen Biotech) was used to construct different recombinogenic DNA fragments according to the manufacturer’s recommendations, with some modifications (Wang et al., 2016). Briefly, amplification of the different recombinogenic DNA fragments was accomplished in two PCR steps (PCR-1 and PCR-2) and one seamless assembly step. The primers were designed to assemble the upstream and downstream regions of the target genes. Forward primers for amplification of the upstream regions and reverse primers for amplification of the downstream regions contained 20 bp overlap regions at their 5′ ends corresponding to the terminal ends of the *Bam*HI-digested vector pSTV28. Reverse primers for amplification of the upstream regions and forward primers for amplification of the downstream regions contained 15–25 bp overlap regions at their 5′ ends. Mutation overlap regions were designed to obtain a specific mutation sequence. In the first step (PCR-1), the upstream and downstream fragments were obtained using their corresponding primers. In the second step (seamless assembly), the products obtained from PCR-1 were mixed with the *Bam*HI-digested vector pSTV28 and assembled using the seamless cloning and assembly kit. The assembled products were confirmed using the forward primers corresponding to the upstream regions and the reverse primers corresponding to the downstream regions of the target genes, with the resulting amplicons subjected to Sanger sequencing. Finally (PCR-2), the specific recombinogenic DNA fragments flanked by the homologous arms (≥40 bp) were obtained using the corresponding primers. Further details on the preparation of various recombinogenic DNA fragments are provided in Supplementary File S1.

### Construction of Plasmid psacBKan

The *Kan* resistance cassette was amplified from pET-30a using primers Kan-*Bam*HI-F and Kan-*Eco*RI-R (containing 20 bp sequence P2 at the 5′ end), and the gel-purified product was digested with *Eco*RI and *Bam*HI. The *sacB* gene cassette (the negative selection marker) was then amplified from plasmid pUC57-*sacB* using primers sacB-F-*Sph*I (containing 20 bp sequence P1 at the 5′ end) and sacB-R-*Bam*HI, and the gel-purified product was digested with *Sph*I and *Bam*HI. The two cassettes were then ligated into *Eco*RI and *Sph*I double-digested pSTV28 using T4 DNA ligase, and the resulting recombinant plasmid, designated psacBKan, was transformed into Trans1-T1 phage-resistant chemically competent *E. coli* (TransGen Biotech).

To confirm that the *sacBKan* cassette could be used for selection, psacBKan was electroporated into wild-type strain JS. Following overnight culture at 37°C, the wild-type and psacBKa*n*-carrying strains were diluted 1:100 in LB medium and cultured at 37°C to logarithmic phase. Aliquots (100 μl) of each of the cultures were then spread onto LB agar plates containing 8% sucrose or kanamycin (50 μg/ml) and incubated at 37°C for 24 h.

### Construction of the *sacBKan*-Containing Intermediate Strains

**FIGURE 1:**
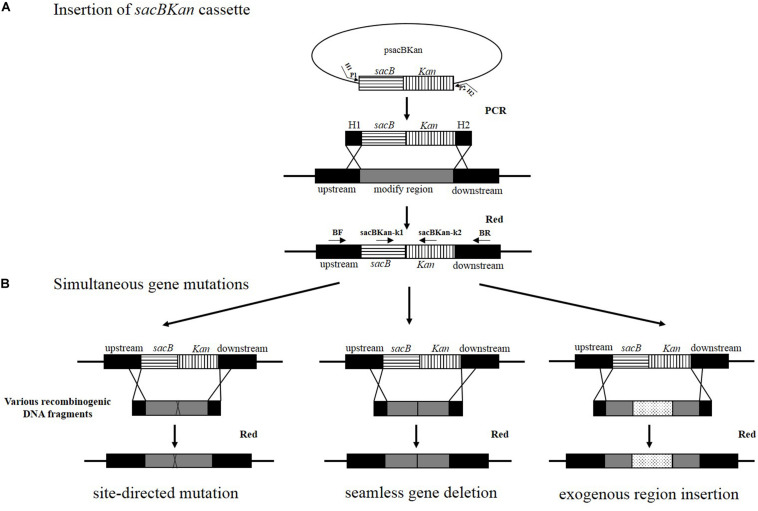
Rationale of the simultaneous gene multiple modifications approach. **(A)** In the first step, the *sacBKan* cassette obtained by using primers H1P1/H2P2 is inserted into the target locus using the red homologous recombination system, and Kan-resistant strains are selected. H1P1/H2P2 included 40- to 50 nt extensions (H1/H2) homologous to regions adjacent to the target-specific locus and 20 bp sequences corresponding to the P1/P2 sequences from psacBKan. Proper *sacBKan* cassette insertion mutant is confirmed by using locus-specific primers (BF and BR) with the corresponding common test primer (psacBKan-k1 or k2) dependent on four reactions by using primer pairs BF/BR, BF/psacBKan-k2, psacBKan-k1/BR, and psacBKan-k1 or k2. **(B)** In the second step, different types of DNA recombinogenic DNA fragments with short homologous arms obtained by using seamless assembly technology are introduced by electroporation to construct site-directed mutation, seamless gene deletion, and exogenous region insertion. Finally, the sucrose-resistant clones are selected on LB plates with 8% sucrose. Positive clones are confirmed by using primer locus-specific primer BF/BR and sequencing.

A linear *sacBKan* fragment was amplified from plasmid psacBKan using the 60–70 bp primers H1P1/H2P2, which included 40–50 bp extensions (H1/H2) homologous to the regions adjacent to the target locus and 20 bp sequences corresponding to the P1/P2 sequences from psacBKan. A 100–200 ng aliquot of gel-purified *sacBKan* was then transformed into electrocompetent *Salmonella* Typhimurium strain JS harboring pKD46 that had been pre-induced for λ red recombinase expression, as described previously (Datsenko and Wanner, 2000). Following electroporation, cells were recovered in 1 ml of SOC medium at 30°C, 200 rpm, for 2 h. Aliquots of the resulting cell suspension were spread onto LB agar plates containing kanamycin (50 μg/ml) to select for kanamycin-resistant clones. Proper insertion of the *sacBKan* cassette was confirmed using locus-specific primers BF and BR together with the corresponding common test primer (psacBKan-k1 or psacBKan-k2), resulting in four separate reactions using primer combinations: BF/BR, BF/psacBKan-k2, psacBKan-k1/BR, and psacBKan-k1/psacBKan-k2 (Figure 1). All confirmed clones were cultured at 30°C to ensure that conventional λ red helper plasmid pKD46 was retained for the second allelic replacement step.

### Simultaneous Generation of Multiple Genetic Mutations

Competent cells prepared for the *sacBKan*-containing intermediate strains harboring pKD46 were induced with L-arabinose. Aliquots (200 ng) of the different recombinogenic DNA fragments were then electroporated into the corresponding intermediate strains. Following electroporation, cells were recovered in 1 ml of SOC medium at 37°C, 200 rpm, for 2 h. Serial dilutions of the bacterial cultures were then spread onto NaCl-minus LB agar plates supplemented with 8% (w/v) sucrose and incubated at 37°C for 24 h to select a sucrose-resistant strain. The correct mutations were confirmed by PCR using locus-specific primers (BF and BR; Figure 1). The recombination efficiencies of the different recombinogenic DNA fragments were assessed based on the proportion of the colonies with correct PCR identification. Finally, the accuracy rate of the gene modifications was further verified by Sanger sequencing.

### Immunodetection Analysis

Immunodetection assays were carried out as described previously (Uzzau et al., 2001). Briefly, strains carrying 3 × FLAG fusion-tagged genes were cultured in LB medium to early stationary phase. Following centrifugation (10,000 × *g*, 5 min), bacterial pellets of 1 ml culture were resuspended in 100 μl of phosphate-buffered saline and mixed with protein loading buffer before being boiled for 10 min and then placed on ice. The resulting lysates were resolved by 15% sodium dodecyl sulfate polyacrylamide gel electrophoresis, transferred to polyvinylidene fluoride membranes, blocked with Tris-buffered saline (pH 8.0) and 3% (v/v) non-fat milk, probed with anti-FLAG M2 monoclonal antibodies (mAbs) (1:1,000) (Sigma), and then incubated with a goat anti-rabbit IgG (whole-molecule) horseradish peroxidase (HRP) conjugate (1:4,000). The FLAG-tagged proteins were visualized using enhanced chemiluminescence.

### Drug Susceptibility Assay

The minimum inhibitory concentrations (MICs) of selected antibiotics against all strains were determined using the twofold broth micro-dilution method according to the Clinical and Laboratory Standard Institute guidelines (Clinical and Laboratory Standards Institute, 2008, 2012). The MICs of amikacin (AMK) and cefuroxime (CXM) were determined independently at least three times.

## Results

### Strategy for Simultaneous Generation of Multiple Genetic Modifications

The principle behind the simultaneous generation of multiple genetic modifications is outlined in Figure 1. The method proposed in the current study included two key steps, which were both mediated by a λ red recombinase-expressing plasmid, pKD46. In the first step, the *sacBKan* cassette amplified from plasmid psacBKan by using primers H1P1/H2P2 was electroporated into a *Salmonella* Typhimurium strain carrying pKD46, and Kan^*r*^ recombinants were selected. The *sacBKan*-containing intermediate strains were then confirmed using locus-specific primers (BF and BR) together with the corresponding common test primer (psacBKan-k1 or k2) (Figure 1A). In the second step, multiple target gene modifications were simultaneously introduced using various homologous recombinogenic DNA fragments to replace the *sacBKan* cassette in the chromosomes of the intermedia strains via allelic exchange (Figure 1B).

**FIGURE 2:**
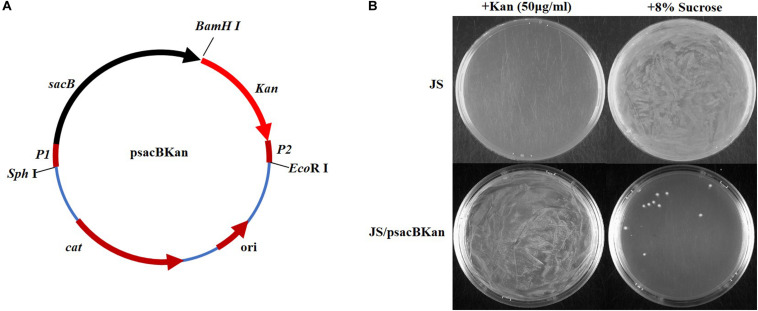
Construction and functional identification of plasmid psacBKan. **(A)** *sacB*, levansucrase-encoding gene is synthetic; Kan, kanamycin-resistant gene results from vector pET30a. P1/P2 sequence fragment is the common template of primers H1P1/H2P2. Other components come from the low-copy-number vector pSTV28 (TaKaRa): cat, chloramphenicol resistance gene; ori, replication origin. **(B)** Functional identification of *scaBKan* cassette on the low-copy-number vector pSTV28. Kanamycin and sucrose sensibility of WT strain JS and the derivative strain JS/psacBKan are identified on LB plate with 50 μg/ml kanamycin or 8% sucrose.

With the proposed method, only one new plasmid (psacBKan) needed to be constructed. This vector was composed of the low-copy-number plasmid pSTV28, a chloramphenicol resistance gene (*Cm*^*r*^), and a *sacBKan* cassette. The *sacBKan* cassette acts as both a positive selection marker (*Kan*^*r*^) and a negative selection marker (*sacB*) and was flanked by 20 bp priming sequences P1 and P2 (Figure 2A). With the use of primers H1P1/H2P2, psacBKan was used as a PCR template to amplify the *sacBKan* cassette flanked by short homologous arms.

To rapidly and efficiently construct different recombinogenic DNA fragments on vector pSTV28, primers were designed to contain both common primer pairs (UF/DR and B^∗^F/B^∗^R) and mutation primer pairs (DF1/UR1, DF2/UR2, DF3/UR3, and DFn/URn; Supplementary Figure S1). Primers UF and DR were designed with a 15–25 bp region of homology to the *Bam*HI-digested pSTV28 sticky end to allow integration of the mutated DNA fragment into the pSTV28 vector. The mutation primers contained 15–25 bp overlapping mutation sequences that were used to introduce specific mutations, including site-directed mutations (Supplementary Figure S1A), seamless gene deletions (Supplementary Figure S1B), and exogenous insertions (Supplementary Figure S1C). Detailed results pertaining to the construction of the various recombinogenic DNA fragments for selected target genes are discussed below.

### Confirmation That the *sacBKan* Cassette Could Be Used for Selection

To confirm that the *sacBKan* cassette could be used for selection, the phenotype of wild-type *Salmonella* Typhimurium strain JS containing the psacBKan plasmid was observed. As shown in Figure 2B, for strain JS, a bacterial lawn was observed on plates containing 8% sucrose, but no colonies were observed on plates containing kanamycin. For strain JS/psacBKan, a bacterial lawn was observed on plates containing kanamycin without sucrose (Figure 2B), while few colonies were observed on plates containing 8% sucrose only. These results were consistent with those obtained for the *sacBKan*-containing intermediate strains (Supplementary Figure S2) and provided evidence that the *sacBKan* cassette could be used for positive/negative selection.

### Construction of Strains Containing Multiple Mutations in *cpxR*

**FIGURE 3:**
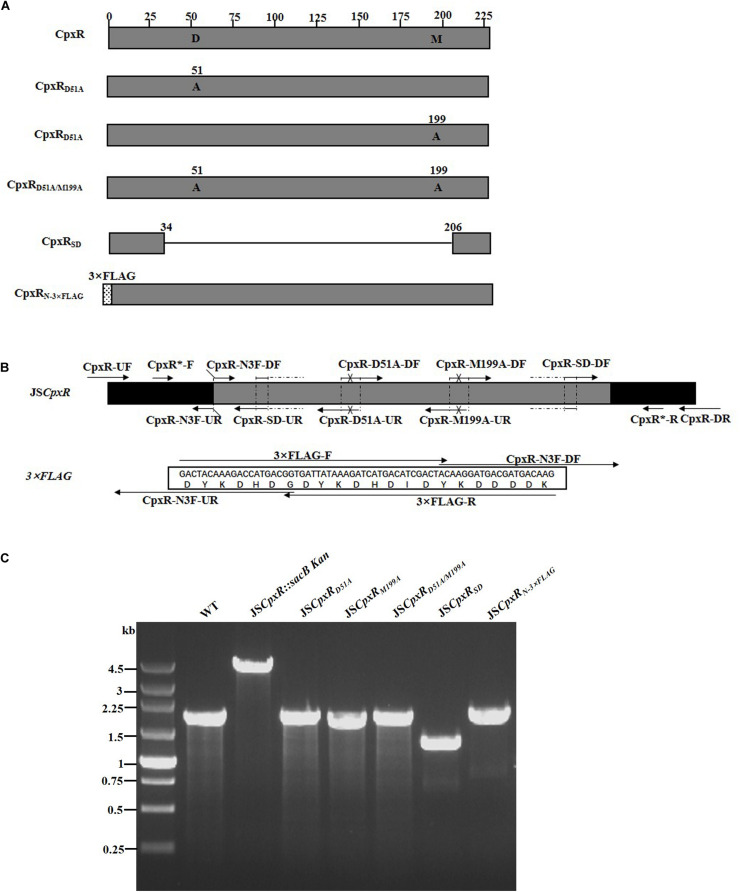
Construction of different gene modifications of *cpxR*gene. **(A)** Schematic diagram of the gene structure of different *cpxR*gene modifications. JS*CpxR*_*D*51*A*_ and JS*CpxR*_*M*199*A*_ mutants include the point mutation at the 51st amino acid (D → A) and at 199th amino acid (M → A) of CpxR protein, respectively; the JS*CpxR*_*D*51*A*/*M*199*A*_ mutant include double-point mutation at 51st and 199th amino acids of the CpxR protein; the JS*CpxR*_*SD*_ mutant was seamlessly deleted in the major region of the CpxR protein. The JS*CpxR*_*N*–3×*FLAG*_ strain expresses N-terminal 3 × FLAG-tagged CpxR protein. **(B)** Primer design for constructing a mutagenic substitution fragment. The universal primers are CpxR-UF/DR and CpxR-F*/R*, and the mutation primers are CpxR-D51A-DF/UR, CpxR-M199A-DF/UR, CpxR-SD-DF/UR, and CpxR-N3F-DF/UR. The overlapping region of CpxR-D51A-DF/UR includes the point mutation at codon 51 (GAC → GCC) of *CpxR* gene used to create the CpxRD51A point mutation fragment. The overlapping region of CpxR-M199A-DF/UR includes the point mutation at codon 199 (ATG → GCG) of the CpxR gene used to create the *CpxR*_*M*199*A*_ point mutation fragment. Both primer pairs CpxR-D51A-DF/UR and CpxR-M199A-DF/UR were used to create the *CpxR*_*D*51*A*/*M*199*A*_ mutation fragment. The overlapping region of CpxR-SD-DF/UR lacks the great mass of codons (34ATG → 206CGC) used to create the *CpxR*_*SD*_ deletion mutation fragment. The primers 3 × FLAG-F/R are two complementary oligonucleotides used to create *3* × *FLAG* tag fragment. The primers CpxR-N3F-DF and CpxR-N3F-DF both include the overlapping region with the 3 × FLAG tag fragment used to create the N-terminal tagging CpxR fragment. **(C)** PCR analysis of CpxR multiple mutants. Band sizes: WT, 1.88 kb; intermediate strain (JS*CpxR:sacBKan*), 4.40 kb; final strain (JS*CpxR*_*D*51*A*_), 1.88 kb; final strain (JS*CpxR*_*M*199*A*_), 1.88 kb; final strain (JS*CpxR*_*D*51*A*/*M*199*A*_), 1.88 kb; final strain (JS*CpxR*_*SD*_), 1.32 kb; final strain (JS*CpxR*_*N*–3 ×*FLAG*_), 1.94 kb. Molecular size markers (250 bp DNA ladder marker, Takara) are indicated. WT, wild strain.

To validate the proposed method, *cpxR* was selected as a target gene to simultaneously construct multiple genetic modifications. CpxR is the response regulator of the Cpx two-component system and consists of an N-terminal receiver domain fused to a C-terminal DNA-binding output domain. Deletion of *cpxR* and mutation of the N-terminal phosphorylation site (D51A) or the C-terminal DNA-binding site (M199A) resulted in inactivation of the Cpx pathway and affected antibiotic resistance (DiGiuseppe and Silhavy, 2003; Mahoney and Silhavy, 2013). In the current study, five different *cpxR* mutants were simultaneously constructed (Figure 3A). In the first step, a *sacBKan* cassette flanked by short homologous arms obtained using the primer pair CpxR-H1P1/CpxR-H2P2 was integrated into the *cpxR* locus using the conventional λ red recombination system to obtain an intermediate strain (JS*cpxR*:*sacBKan/pKD46*). Positive clones were screened by PCR using primer pairs CpxR-F/R (4.4 kb), CpxR-F/sacBKan-k2 (3.1 kb), sacBKan-k1/CpxR-R (3.09 kb), and sacBKan-k1/k2 (1.79 kb) (Supplementary Figure S3).

In PCR-1, different upstream and downstream regions of *cpxR* were amplified using each locus-specific primer, as indicated in Supplementary Table S1 and Figure 3B. In the seamless assembly step, each modified DNA fragment, in which the upstream and corresponding downstream regions were combined, was cloned into *Bam*HI-digested pSTV28 using the seamless assembly system. In PCR-2, different recombinogenic DNA fragments were amplified using the common primer pair CpxR^∗^-F/R. In all cases, the sizes of the PCR products obtained during construction of the various recombinogenic DNA fragments were consistent with the predicted product sizes (Supplementary Figure S4).

**FIGURE 4:**
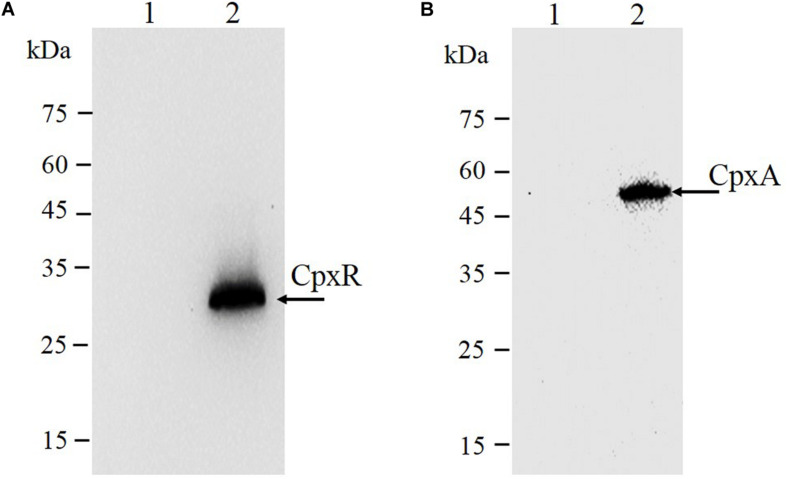
Immunodetection of 3 × FLAG epitope-tagged protein. Tagged bacterial lysates were subjected to electrophoretic separation in a 12% SDS-PAGE gel and then transferred to polyvinylidene fluoride (PVDF) membranes and probed with anti-FLAG M2 mAbs (Sigma). **(A)** CpxR protein: 1, strain JS (parent strain); 2, strain JS*CpxR*_*N*–3 ×*FLAG*_. The expected molecular mass of 3×FLAG-CpxR is 28.99 kDa. **(B)** CpxA protein: 1, strain JS (parent strain); 2, strain JS*CpxA*_*C*–3 ×*FLAG*_. The expected molecular mass of CpxA-3 × FLAG is 54.32 kDa. On the left are protein marker positions and molecular length (kDa).

As shown in Figure 3C, the second λ red recombination step, in which each target recombinogenic DNA fragment replaced the *sacBKan* cassette, was confirmed by PCR. PCR-based analysis of the genomic DNA of each mutant strain using primers CpxR-F and CpxR-R produced fragments of approximately 1.88 kb (*cpxR*_*D*51*A*_, *cpxR*_*M*199*A*_, and *cpxR*_*D*51*A*/M199*A*_), 1.32 kb (*cpxR*_*SD*_), and 1.94 kb (*cpxR*_*N*–3×*FLAG*_). In more than 96% of cases, the sizes of the obtained fragments were in agreement with the predicted sizes of the fragments containing the corresponding modification (Table 2). Sanger sequencing of each mutant confirmed that the accuracy rate of gene modification was >90% (Table 2). To examine the effects of each c*pxR* mutation on the resistance of *Salmonella* Typhimurium to aminoglycoside antibiotics, the MICs of amikacin and streptomycin for each mutant strain were determined and compared to those of the wild-type strain. As shown in Table 3, except for strains JS*CpxR*_*M*199*A*_ and JS*CpxR*_*N*–3 ×*FLAG*_, 2-4-fold decreases in the MICs of AMK and CXM, respectively, were observed for all strains. Finally, tagged recombination mutant JS*CpxR_*N*–3×*FLAG*_* was visualized by western blotting using anti-FLAG monoclonal antibody as the primary antibody. As shown in Figure 4A, the anti-FLAG monoclonal antibody recognized the predicted 3 × FLAG-CpxR fusion product (28.99 kDa).

**TABLE 2:** Efficiency of gene modification by the proposed method.

Modification	No. of strains tested by PCR	Modification rate (%)		No. of strains tested by PCR fragment sequencing	Modification rate (%)	
		Fault	Correct		Fault	Correct
CpxR_*D*51*A*_	30	1 (3.3)	29 (96.7)	10	0 (0)	10 (100)
CpxR_*M*199*A*_	30	1 (3.3)	29 (96.7)	10	0 (0)	10 (100)
CpxR_*D*51*A/M*199*A*_	30	1 (3.3)	29 (96.7)	10	1 (10)	9 (90)
CpxR_*SD*_	30	0 (0)	30 (100)	10	0 (0)	10 (100)
CpxR_*C–*3×*FLAG*_	30	0 (0)	30 (100)	10	0 (0)	10 (100)
CpxA_*L*38*F*_	30	1 (3.3)	29 (96.7)	10	0 (0)	10 (100)
CpxA_Δ 92–104_	30	0 (0)	30 (100)	10	1 (10)	9 (90)
CpxA_*C–*3×*FLAG*_	30	2 (6.7)	28 (93.3)	10	0 (0)	10 (100)
acrB_*D*408*A*_	30	2 (6.7)	28 (93.3)	10	0 (0)	10 (100)
acrB_*SD*_	30	0 (0)	30 (100)	10	1 (10)	9 (90)

**TABLE 3:** Susceptibility of the *Salmonella* mutants to antibiotics.

Strains	MICs (μ g/ml)	
	AMK	CXM
JS	2	4
JS*CpxR_*D*51*A*_*	0.5	2
JS*CpxR_*M*199*A*_*	2	4
JS*CpxR_*D*51*A/M*199*A*_*	0.5	2
JS*CpxR*_*SD*_	0.5	2
JS*CpxR_*N–*3×*FLAG*_*	2	4
JS*CpxA_*L*38*F*_*	8	16
JS*CpxA*_Δ 92–104_	8	16
JS*CpxA_*C–*3×*FLAG*_*	2	4
JS*acrB_*D*408*A*_*	0.5	0.13
JS*acrB*_*SD*_	0.5	0.13

*AMK, Amikacin; CXM, Cefuroxime.*

### Application of the Proposed Method in Other Genes

To demonstrate the applicability of the proposed method in other genes, *cpxA* and *acrB* were selected as target genes. Both genes are closely associated with antibiotic resistance in *Salmonella* Typhimurium (Baucheron et al., 2004; Huang et al., 2016; Wang-Kan et al., 2017).

**FIGURE 5:**
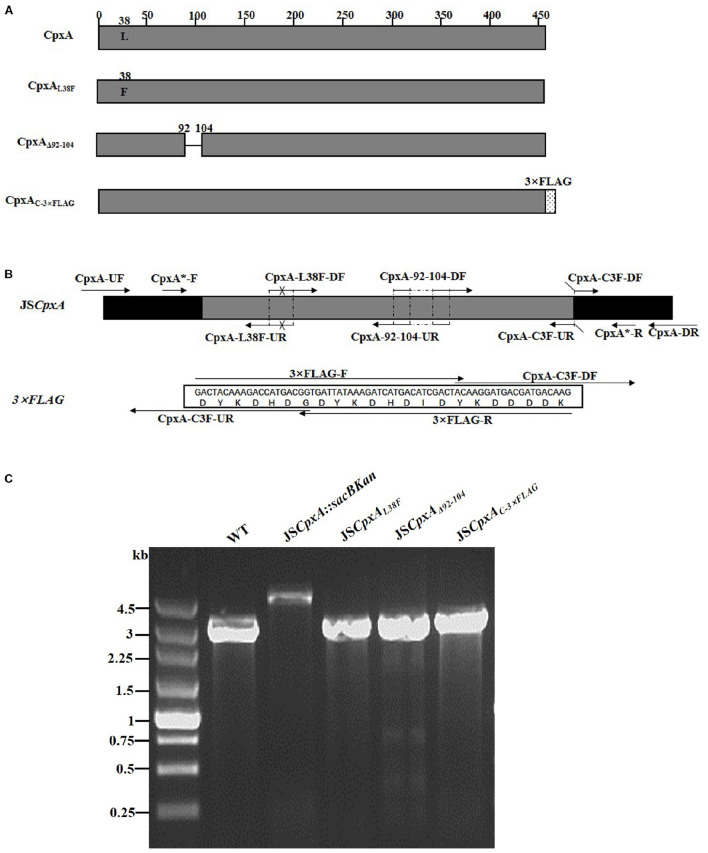
Construction of different gene modifications of *cpxA* gene. **(A)** Schematic diagram of the gene structure of different *cpxA* gene modifications. The JS*CpxA*_*L*38*F*_ mutant includes the point mutation at the 38th amino acid (L → F) of the CpxA protein. The JS*CpxA*_Δ 92–104_ mutant lacks a region including 13 amino acids encoding the periplasmic loop of the CpxA protein. The JS*CpxA*_*C*–3×*FLAG*_ strain expresses the C-terminal 3 × FLAG-tagged CpxA protein. **(B)** Primer design for constructing the mutagenic substitution fragment. The universal primers are CpxA-UF/DR and CpxA-F*/R*, and the mutation primers are CpxA-L38F-DF/UR, CxpA-92-104-DF/UR, and CpxA-C3F-DF/UR. The overlapping region of CpxA-L38F-DF/UR includes the point mutation at codon 38 (CTG → TTT) of the CpxA gene used to create the *CpxA*_*L*38*F*_ point mutation fragment. The overlapping region of CpxA-92-104-DF/UR lacks 13 codons (92GGA → 104ATC) used to create the *CpxA*_Δ 92–104_ deletion mutation fragment. The primers 3 × FLAG-F/R are two complementary oligonucleotides used to create the 3 × FLAG tag fragment. The primers CpxA-C3F-DF and CpxA-C3F-DF both include the overlapping region with the 3 × FLAG tag fragment used to create the C-terminal tagging CpxA fragment. **(C)** PCR analysis of CpxA multiple mutants. Band sizes: WT, 2.89 kb; intermediate strain (JS*CpxA*:*sacBKan*), 4.76 kb; final strain (JS*CpxA*_*L*38*F*_), 2.89 kb; final strain (JS*CpxA*_Δ 92–104_), 2.85 kb; final strain (JS*CpxA*_*C*–3×*FLAG*_), 2.95 kb. Molecular size markers (250 bp DNA ladder marker, Takara) are indicated. WT, wild strain.

CpxA is the histidine sensor kinase/phosphatase of the Cpx two-component system (Jianming et al., 1993). *cpxA* variants encoding a CpxA protein missing amino acids 92–104 or containing site-specific mutation L38F constitutively induced activation of the Cpx system, resulting in increased antibiotics resistance (Raivio and Silhavy, 1997; Humphreys et al., 2004). To generate different *cpxA* mutants (Figure 5A), the intermediate strain was constructed by integrating a *sacBKan* cassette obtained using primers CpxA-H1P1/CpxA-H2P2 into the *cpxA* locus using the λ red recombination system. PCR-based analysis of the genomic DNA of the intermediate strain was then conducted using primer pairs CpxA-F/R (4.76 kb), CpxA-F/sacBKan-k2 (3.23 kb), sacBKan-k1/CpxA-R (3.32 kb), and sacBKan-k1/k2 (1.79 kb) (Supplementary Figure S3). The various recombinogenic DNA fragments were then amplified using common primer pair CpxA^∗^-F/R from the corresponding chimeric plasmids constructed using the seamless assembly system (Figure 5B). The sizes of the PCR products obtained during construction of various recombinogenic DNA fragments were consistent with the predicted product sizes (Supplementary Figure S5). As shown in Figure 5C, the second λ red recombination event was confirmed by PCR. PCR-based analysis of the genomic DNA of each mutant was conducted using primers CpxA-F and CpxA-R, resulting in fragments of approximately 2.89 kb (*cpxA*_*L*38*F*_), 2.85 kb (*cpxA*_Δ 92–104_), and 2.95 kb (*cpxA*_*C*–3×*FLAG*_). A mutation success rate >93% was achieved for *cpxA*, as determined by PCR analysis, while Sanger sequencing confirmed that the accuracy rate of the gene modifications was >90% (Table 2). The effects of the *cpxA* mutations on antibiotic resistance were then assessed. As shown in Table 3, except for strain JS*CpxA*_*C*–3 ×*FLAG*_, 4-fold increases in the MICs of AMK and CXM were observed for all mutant strains compared with the wild type. Finally, tagged recombination mutant JS*CpxA*_*C*–3×*FLAG*_ was visualized by western blotting. As shown in Figure 4B, the anti-FLAG monoclonal antibody recognized the predicted fusion product CpxA-3 × FLAG (54.32 kDa).

**FIGURE 6:**
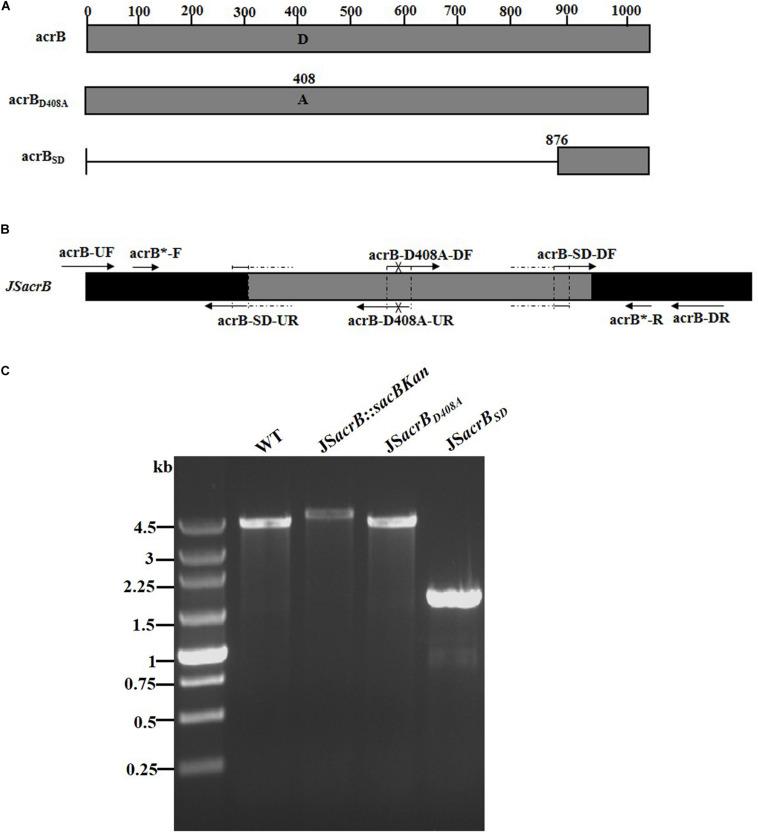
Construction of different gene modifications of *acrB* gene. **(A)** Schematic diagram of the gene structure of different acrB gene modifications. The JS*acrB*_*D*408*A*_ mutant includes the point mutation at the 408th amino acid (A → D) of the acrB protein. The JS*acrB*_*SD*_ mutant was seamlessly deleted in the major region of the acrB protein. **(B)** Primer design for constructing the mutagenic substitution fragment. The universal primers are acrB-UF/DR and acrB-F*/R*, and the mutation primers are acrB-M408A-DF/UR and acrB-SD-DF/UR. The overlapping region of acrB-D408A-DF/UR includes the point mutation at codon 408 (GAC → GCC) of the acrB gene used to create the *acrB*_*D*408*A*_ point mutation fragment. The overlapping region of acrB-SD-DF/UR lacks a great mass of codons (1ATG → 876CTG) used to create the *acrB*_*SD*_ deletion mutation fragment. **(C)** PCR analysis of *acrB* multiple mutants. Band sizes: WT, 4.19 kb; intermediate strain (JS*acrB*:*sacBKan*), 4.78 kb; final strain (JS*acrB*_*D*408*A*_), 2.19 kb; final strain (JS*acrB*_*SD*_), 1.56 kb. Molecular size markers (250 bp DNA ladder marker, Takara) are indicated. WT, wild strain.

AcrB is a component of the well-characterized AcrAB-TolC multidrug efflux pump system and directs efflux-mediated multidrug resistance in *Salmonella* Typhimurium (Baucheron et al., 2004; Wang-Kan et al., 2017; Weston et al., 2017). Deletion of *arcB* or modification to introduce a D408A substitution conferred susceptibility to AcrB substrates by abolishing efflux activity (Wang-Kan et al., 2017). In the current study, we successfully undertook site-directed mutagenesis and seamless deletion of *acrB* using the proposed system (Figure 6A). PCR-based analysis of the genomic DNA of the intermediate strain was then conducted using primer pairs acrB-F/R (4.78 kb), acrB-F/sacBKan-k2 (3.46 kb), sacBKan-k1/acrB-R (3.11 kb), and sacBKan-k1/k2 (1.79 kb) (Supplementary Figure S3). Different recombinogenic DNA fragments were amplified by PCR using the common primer pair acrB^∗^-F/R from the corresponding chimeric plasmids constructed using the seamless assembly system (Figure 6B). The sizes of PCR products obtained during construction of the recombinogenic DNA fragments were consistent with the predicted product sizes (Supplementary Figure S6). As shown in Figure 6C, the second λ red recombination event was confirmed by PCR. PCR-based analysis of the genomic DNA of each mutant using primers acrB-F and acrB-R resulted in fragments of approximately 4.18 kb (*acrB*_*D*408*A*_) and 1.56 kb (*acrB*_*SD*_). The success rate of mutations in *acrB* was >93%, as determined by PCR, while Sanger sequencing confirmed that the accuracy rate for the genetic modifications was >90% (Table 2). The effects of the *acrB* mutations on the antibiotic resistance were then assessed. As shown in Table 3, 4- and 32-fold decreases in the MICs of AMK and CXM, respectively, were observed for both mutant strains compared with the wild-type strain.

## Discussion

In this study, we developed a flexible method for the simultaneous generation of multiple variations at the same locus in the genome of *Salmonella* Typhimurium. The genetic modification strategy combined an intracellular recombination system (λ red-mediated recombination), counterselection marker *sacB*, and seamless cloning and assembly *in vitro*. The proposed method allows easy and effective simultaneous integration of various vector-based modification alleles into the corresponding locus of the *Salmonella* Typhimurium chromosome using only conventional λ red recombination protocols (Datsenko and Wanner, 2000).

The method developed in this study couples the λ red recombination system with counterselection markers and uses two separate rounds of recombination. Similar strategies have been proposed previously (Gerlach et al., 2009; Stringer et al., 2012); however, neither of the earlier methods avoids the tedious process of obtaining various recombinogenic DNA fragments or overcome the limitation of short synthetic oligonucleotide length. Construction of a suitable homologous recombinogenic fragment is a key step in the genetic modification process. Traditional homologous fragment construction methods tend to involve digestion and ligation, overlap extension PCR, or complementary primers (Gerlach et al., 2009; Wei et al., 2010; Geng et al., 2016). However, digestion and ligation-based methods are not suitable for generating seamless products, and the alternative methods are inefficient for the construction of multiple mutations and the assembly of multiple DNA fragments. The method described in the current study overcomes these problems by using enzymatic assembly strategies that involve overlapping DNA fragments. With the use of a one-step isothermal reaction, various DNA fragments sharing terminal sequence overlaps are integrated into a single target DNA fragment (Gibson, 2011; Moradpour and Abdulah, 2017). Through an *in vitro* recombinant enzyme system, various types of specific DNA fragments can be constructed to produce seamless mutants, single and multipoint substitution mutants, and fusion genes. With the use of universal primers and a pair of mutation primers, a specific DNA modification fragment can rapidly be constructed. This method ensures a low error rate during cloning and sequencing of the designed DNA fragment and avoids the risk of mutation that accompanies long primer synthesis and other unknown factors. We note that in some cases, DNA substitution fragments were not directly amplified from extracted plasmids using primers B^∗^F/B^∗^R but were subjected to an initial round of amplification using vector universal primers RV-M and M13-47 to avoid interference from the *E. coli* genome, especially for point mutations. This is because of the high level of homology between the genomes of *Salmonella* Typhimurium and *E. coli*, which was used for vector construction. This precautionary step ensures the sequence accuracy of the target DNA substitution fragment, which can be confirmed by direct PCR amplification and sequencing from the bacterial suspension. Thus, this strategy can be used to efficiently assemble different DNA fragments on a vector and construct synthetic and natural genes as well as being a useful molecular engineering tool (Gibson, 2011).

The proposed method also provides flexibility in designing and introducing different mutations at the same locus. In the current study, by coupling different overlapping primer pairs, compound DNA modifications were also constructed. As shown by the construction of double-point mutation fragment *CpxR*_*D*51*A/M*199*A*_ (Supplementary Figure S4), a suitable recombinogenic DNA fragment can be obtained using primer pairs designed for the construction of single-point mutation fragments in tandem (e.g., *cpxR*_*D*51*A*_ and *cpxR*_*M*199*A*_), eliminating the need to design additional primers. Although the intermedia strains have to be constructed using two separate rounds of recombination strategy, the design of the universal primers makes it easy not only to simultaneously construct and identify different mutagenesis events at a same locus but also to move mutations from one strain into another using the *sacBKan*-containing strains. It is important for the functional analysis of target genes to introduce the mutant alleles from different strains into a wild-type strain (Hayashi and Tabata, 2013). The mutant allele confirmation is conducted by PCR and Sanger sequencing so that the PCR products obtained using universal primers B^∗^F and B^∗^R can be directly integrated into the target locus using the λ red recombination system.

An applicable selective marker is a critical parameter in the second step of homologous recombination. In previous studies (Gerlach et al., 2009; Stringer et al., 2012; Yang et al., 2014; Pyne et al., 2015), a counterselection marker such as *thyA* or *tetA* could only be used in host strains with a corresponding gene deletion. Several more efficient positive selection markers, including the I-*Sce*I endonuclease and the CRISPR/Cas9 system, have also been used, but methods using two or more helper and donor vectors make it difficult to cure plasmids from the resulting strains. Our method only uses the temperature-sensitive helper plasmid pKD46, avoiding the need to cure the cells of multiple helper plasmids. *sacB* is a common counterselection marker used in Gram-negative bacterial species, including *Salmonella* Typhimurium (Kang et al., 2002b; Zhang et al., 2002; Gal-Mor et al., 2008). Importantly, it does not share a high degree of homology with sequences in the *Salmonella* genome and using *sacB* from *Bacillus subtilis* as a counterselection marker ensures commonality. Our results also confirmed that *sacB* is sufficiently efficient for the counterselection of mutations in *Salmonella* Typhimurium.

Suitable fusion strategies ensure the accuracy of the experimental results (Einhauer and Jungbauer, 2001). To date, many effective protein C-terminal epitope-tagging methods have been established (Chiang and Rubin, 2002; Kaltwasser et al., 2002; Cho et al., 2006); however, there are few reports on N-terminal tagging methods, which is due to the N-terminal fusion between the target gene and an epitope-encoding sequence needing to avoid the introduction of a marker or scar. For some proteins, N-terminal tagging will be also more effective and specifically adapted because C-terminal tagging can produce a polar effect on downstream protein expression. For example, in the Cpx system, the initiation codon of the *cpxA* gene is within the N-terminus region of the *cpxR* gene, so C-terminal tagging of CpxR will inevitably cause a polar effect on CpxA. In our system, effective N-terminal tagging is used to circumvent these pitfalls. We initially attempted to tag CpxR and CpxA with a 1 × FLAG tag; however, western blotting revealed that the resulting tagged *Salmonella* proteins were not detected with anti-FLAG mAbs (results not shown), which is consistent with previous findings (Uzzau et al., 2001). We then replaced the 1 × FLAG epitope with the more sensitive 3 × FLAG epitope (Hernan et al., 2000), and as expected, the resulting 3 × FLAG-tagged proteins were more easily detected. We therefore predict that tagging with other protein tags such as hemagglutinin peptides, 6 × His, and c-Myc is likely to be easily accomplished using our method.

In summary, we developed a flexible and simple method for the simultaneous generation of multiple genetic modifications, including site-directed mutations, seamless gene deletions, and N- and C-terminal epitope tagging, at the same locus in *Salmonella* Typhimurium. This efficient method will aid in the functional analysis of bacterial genes and in vaccine development by allowing the modification of endogenous and even exogenous genes. Because of the successful use of the λ red homologous recombination system and the counterselection marker *sacB* in other bacterial species (Tan et al., 2012; Hmelo et al., 2015; Oh et al., 2015), it is likely that this strategy will prove effective in many species in addition to *Salmonella* Typhimurium.

## Data Availability Statement

The original contributions presented in the study are included in the article/Supplementary Material, further inquiries can be directed to the corresponding author/s.

## Author Contributions

WJ, JL, and SW designed the experiment. QC and YL were responsible for funding acquisition and project supervision. WJ, XL, and YL contributed to manuscript writing. All authors contributed to the article and approved the submitted version.

## Conflict of Interest

The authors declare that the research was conducted in the absence of any commercial or financial relationships that could be construed as a potential conflict of interest.

## Supplementary Material

FIGURE S1 Construction of mutagenic substitution fragment. In this study, different types of DNA mutagenic substitution fragments are constructed by seamless cloning and assembly system and matched PCR. Firstly, short fragments with homologous ends are created by colony PCR. Then, short fragments are integrated into the clone vector pSTV28. Finally, integrated mutagenic substitution fragment is created by colony PCR. Design primers conclude universal primers (UF/DR and F^∗^/R^∗^) and mutation primers (F1/R1, F2/R2, and F3/R3). For correct assemble, mutation primer pairs and UF/DR contain overlapping region at 5′ ends and extension region at the 3′ ends. UF/DR have homologous overlapping region (GAA—GGG/TCC—GAG) of *Bam*HI site of clone vector pSTV28 used to integrate mutagenic substitution fragment into the clone vector. Every mutation primer pairs have specific mutation on overlapping region used to assembly designed gene modification. Using primers F1/R1, point mutation fragment is constructed **(A)**. Using primers F2/R2, seamless gene deletion fragment is constructed **(B)**. Using primers F3/R3, exogenous region insertion fragment is constructed **(C)**. Finally, using primer pairs F^∗^/R^∗^, PCR products are generated including short homology extension of two flanks of modify gene.

FIGURE S2 Functional identification of *scaBKan* cassette inserting some specific locus. Kanamycin and sucrose sensibility of intermedia strains JS*CpxR:sacBKan*, JS*CpxA:sacBKan*, and JS*acrB:sacBKan* are identified on LB plate with 50 μg/ml kanamycin and 8% sucrose.

FIGURE S3 PCR for *sacBKan* cassette integration. Intermediate strain JS*CpxR:sacBKan*: CpxR-F/R, 4.40 kb; CpxR-F/sacBKan-k2: 3.10kb; sacBKan-k1/CpxR-R: 3.09 kb; sacBKan-k1/k2: 1.79 kb. Intermediate strain JS*CpxA:sacBKan*: CpxA-F/R, 4.76 kb; CpxA-F/sacBKan-k2: 3.23 kb; sacBKan-k1/CpxA-R: 3.31 kb; sacBKan-k1/k2: 1.79 kb. Intermediate strain JS*acrB:sacBKan*: acrB-F/R, 4.78 kb; acrB-F/sacBKan-k2: 3.46 kb; sacBKan-k1/acrB-R: 3.11 kb, sacBKan-k1/k2: 1.79 kb.

FIGURE S4 Preparation of various recombinogenic DNA of *CpxR*. In PCR-1, the corresponding downstream and upstream regions of various DNA modification fragments *CpxR*_*D*51*A*_, *CpxR*_*M*199*A*_, *CpxR*_*SD*_, *CpxR*_*N*–3×*FLAG*_, and *CpxR*_*D*51*A*/*M*199*A*_ (require additional midstream), were amplified by PCR using the genomic DNA of WT strain JS. 3 × FLAG, tagging the N-terminal of CpxR, was amplified by PCR using a pairs of complementary oligonucleotides. UP, upstream region of the target gene to be modified. DW, downstream region of the target gene to be modified. MD, midstream region of the target gene to be modified. In seamless assembly, successful assembly of corresponding PCR products were confirmed by PCR with primers CpxR-UF/DR. In PCR-2, various recombinogenic DNA fragments were amplified by PCR with primers CpxR^∗^-F/R. Molecular size markers (DL2000 DNA marker, Takara) are indicated.

FIGURE S5 Preparation of various recombinogenic DNA of *CpxA*. In PCR-1, the corresponding downstream and upstream regions of various DNA modification fragments *CpxA*_*L*38*F*_, *CpxA*_Δ 92–104_ and *CpxA*_*C*–3×*FLAG*_, were amplified by PCR using the genomic DNA of WT strain JS. 3 × FLAG, tagging the C-terminal of CpxA, was amplified by PCR using a pair of complementary oligonucleotides. UP, upstream region of the target gene to be modified. DW, downstream region of the target gene to be modified. In seamless assembly, successful assembly of corresponding PCR products were confirmed by PCR with primers CpxA-UF/DR. In PCR-2, various recombinogenic DNA fragments were amplified by PCR with primers CpxA^∗^-F/R. Molecular size markers (250 bp DNA ladder marker, Takara) are indicated.

FIGURE S6 Preparation of various recombinogenic DNA of *acrB*. In PCR-1, the corresponding downstream and upstream regions of various DNA modification fragments, *acrB*_*D*408*A*_ and *acrB*_*SD*_ were amplified by PCR using the genomic DNA of WT strain JS. UP, upstream region of the target gene to be modified. DW, downstream region of the target gene to be modified. In seamless assembly, successful assembly of corresponding PCR products were confirmed by PCR with primers acrB-UF/DR. In PCR-2, various recombinogenic DNA fragments were amplified by PCR with primers acrB^∗^-F/R. Molecular size markers (DL2000 DNA marker, Takara) are indicated.

TABLE S1 Primers used in this study.

FILE S1 Preparation of various recombinogenic DNA fragments.
