# Supplementary figures and images for: Development of a Method for Simultaneous Generation of Multiple Genetic Modification in Salmonella enterica Serovar Typhimurium
Source: Front Genet. 2020 Sep 24;11:563491. doi: 10.3389/fgene.2020.563491 (PMC7544003; doi:10.3389/fgene.2020.563491)

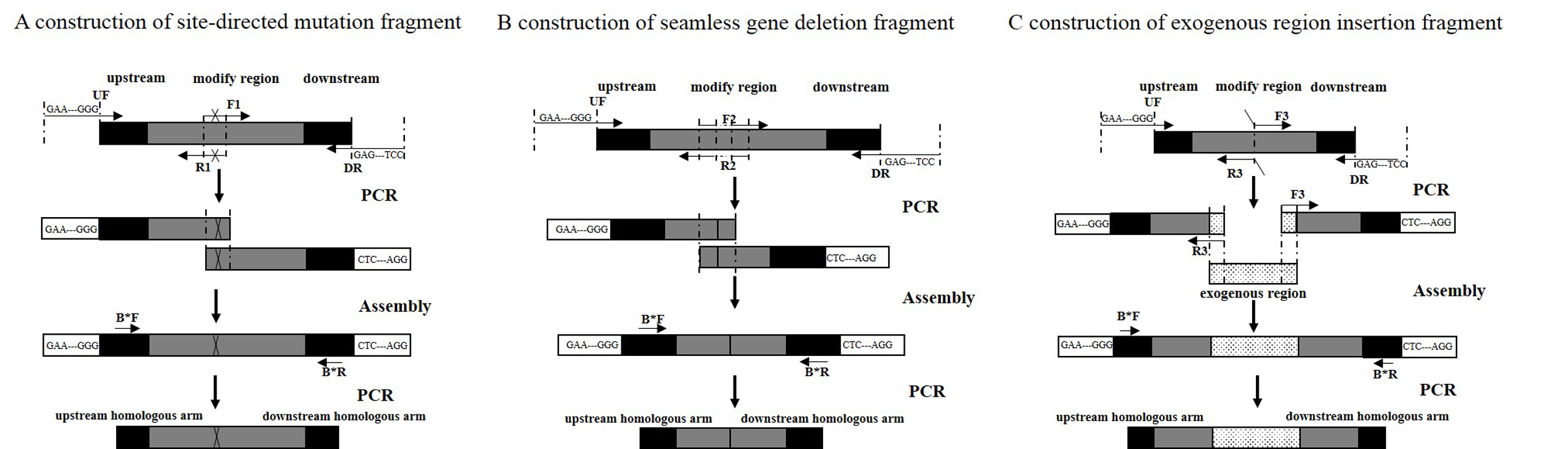

Supplement: FIGURE S1 — Construction of mutagenic substitution fragment. In this study, different types of DNA mutagenic substitution fragments are constructed by seamless cloning and assembly system and matched PCR. Firstly, short fragments with homologous ends are created by colony PCR. Then, short fragments are integrated into the clone vector pSTV28. Finally, integrated mutagenic substitution fragment is created by colony PCR. Design primers conclude universal primers (UF/DR and F∗/R∗) and mutation primers (F1/R1, F2/R2, and F3/R3). For correct assemble, mutation primer pairs and UF/DR contain overlapping region at 5′ ends and extension region at the 3′ ends. UF/DR have homologous overlapping region (GAA—GGG/TCC—GAG) of BamHI site of clone vector pSTV28 used to integrate mutagenic substitution fragment into the clone vector. Every mutation primer pairs have specific mutation on overlapping region used to assembly designed gene modification. Using primers F1/R1, point mutation fragment is constructed (A). Using primers F2/R2, seamless gene deletion fragment is constructed (B). Using primers F3/R3, exogenous region insertion fragment is constructed (C). Finally, using primer pairs F∗/R∗, PCR products are generated including short homology extension of two flanks of modify gene. [file Image_1.TIF]

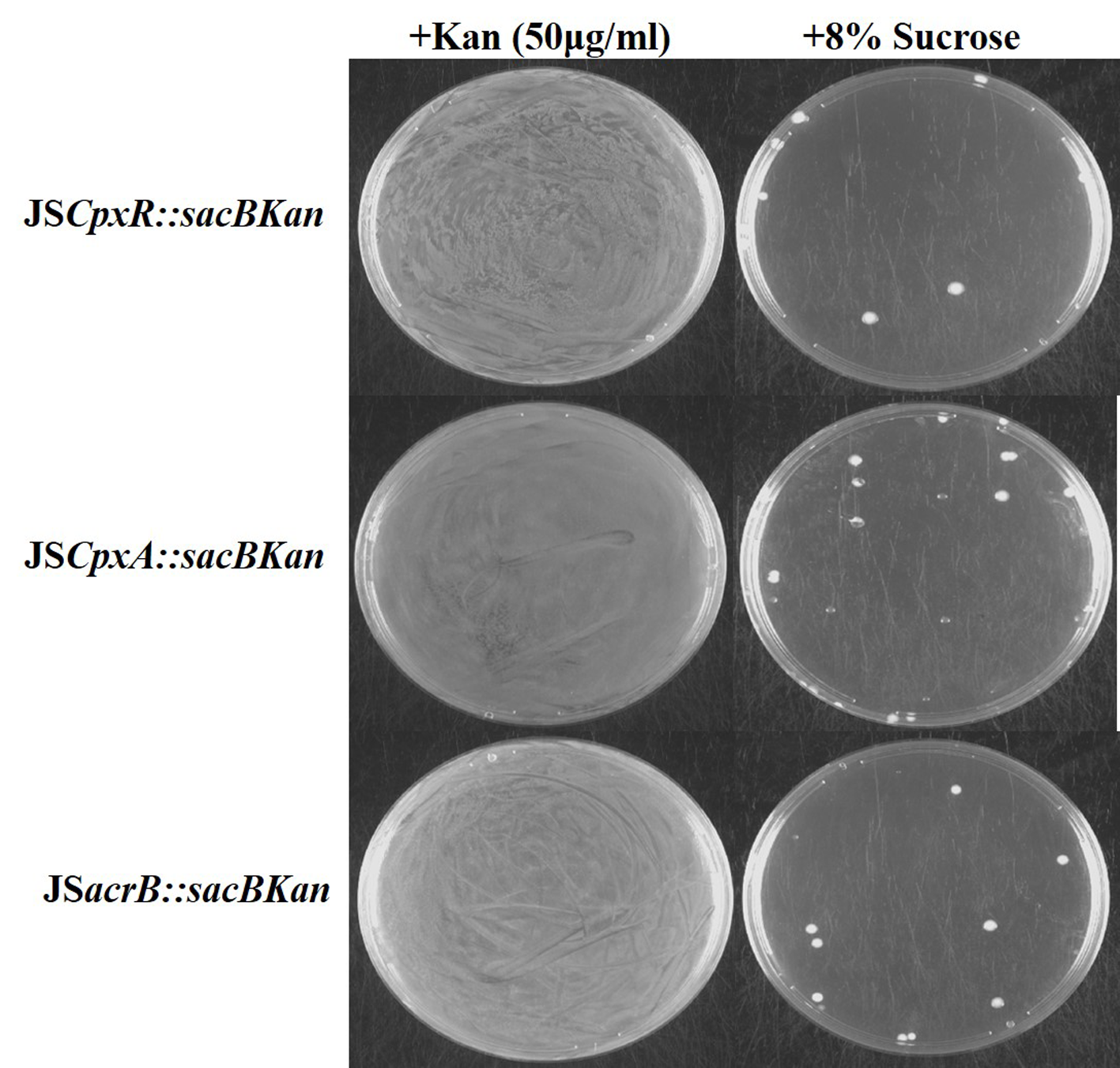

Supplement: FIGURE S2 — Functional identification of scaBKan cassette inserting some specific locus. Kanamycin and sucrose sensibility of intermedia strains JSCpxR:sacBKan, JSCpxA:sacBKan, and JSacrB:sacBKan are identified on LB plate with 50 μg/ml kanamycin and 8% sucrose. [file Image_2.TIF]

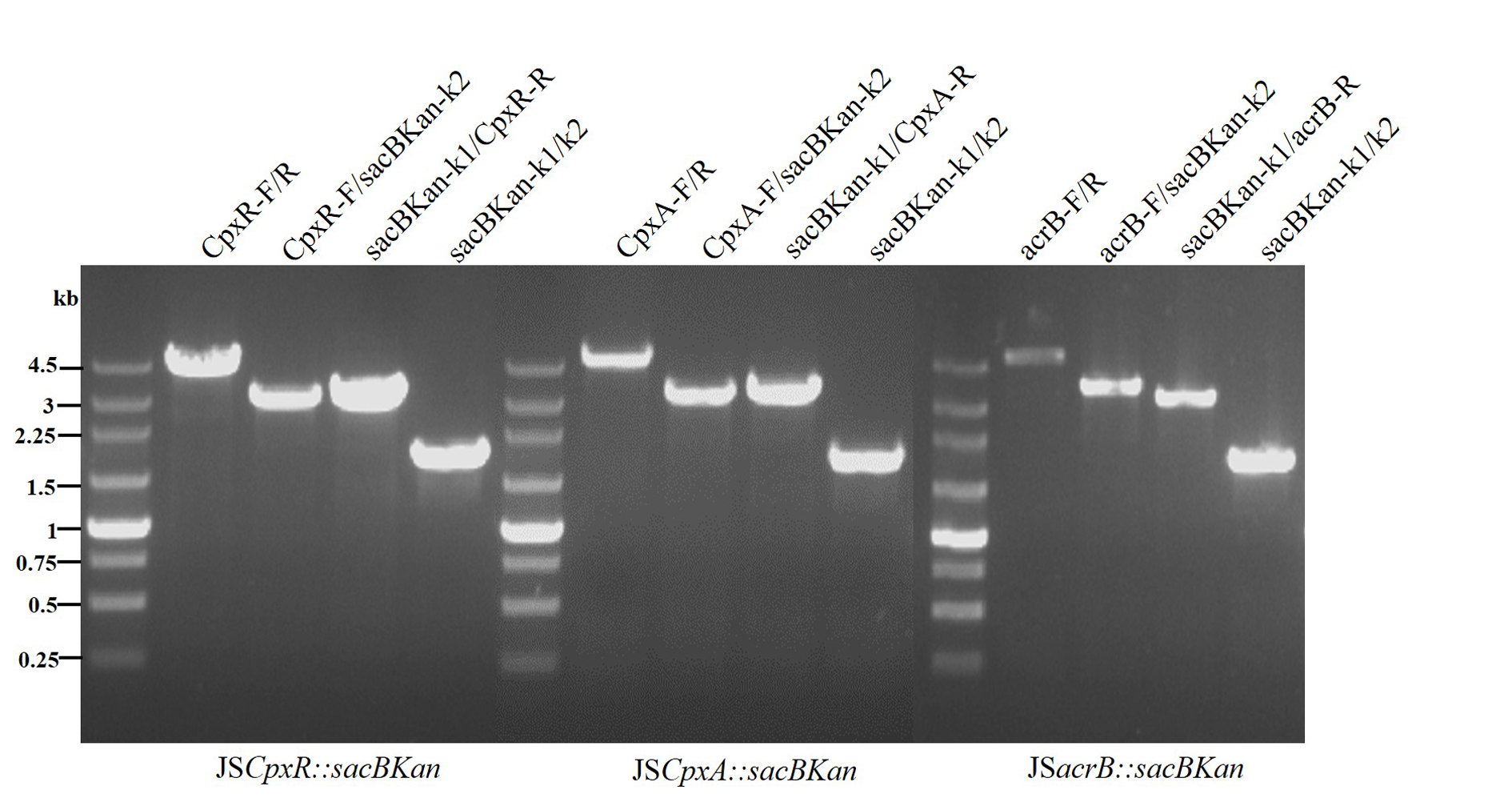

Supplement: FIGURE S3 — PCR for sacBKan cassette integration. Intermediate strain JSCpxR:sacBKan: CpxR-F/R, 4.40 kb; CpxR-F/sacBKan-k2: 3.10kb; sacBKan-k1/CpxR-R: 3.09 kb; sacBKan-k1/k2: 1.79 kb. Intermediate strain JSCpxA:sacBKan: CpxA-F/R, 4.76 kb; CpxA-F/sacBKan-k2: 3.23 kb; sacBKan-k1/CpxA-R: 3.31 kb; sacBKan-k1/k2: 1.79 kb. Intermediate strain JSacrB:sacBKan: acrB-F/R, 4.78 kb; acrB-F/sacBKan-k2: 3.46 kb; sacBKan-k1/acrB-R: 3.11 kb, sacBKan-k1/k2: 1.79 kb. [file Image_3.TIF]

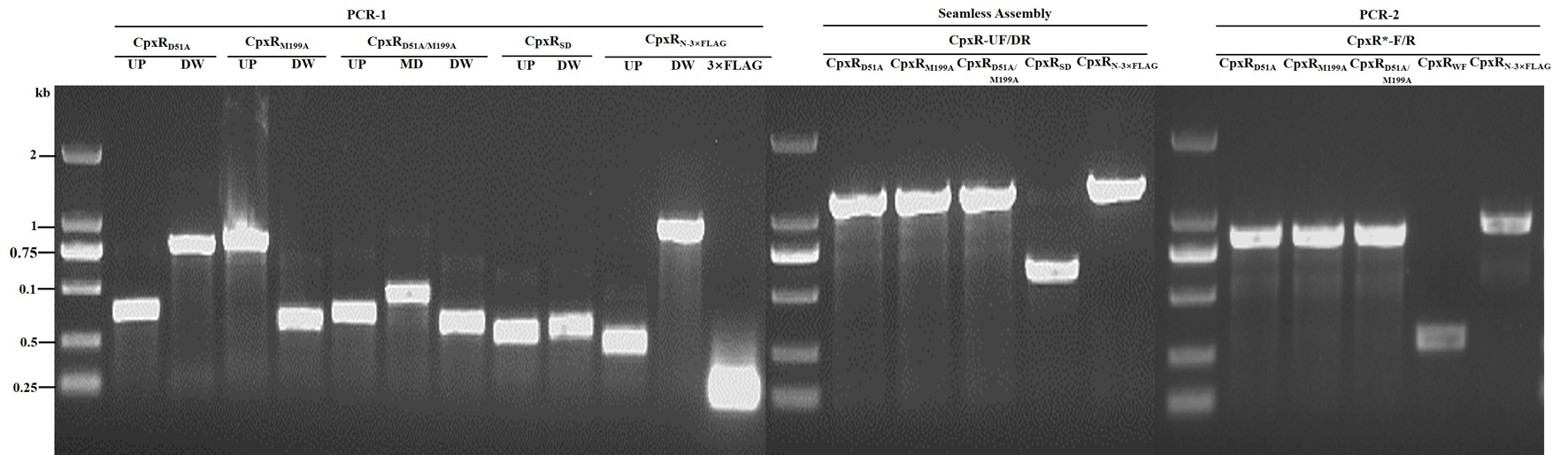

Supplement: FIGURE S4 — Preparation of various recombinogenic DNA of CpxR. In PCR-1, the corresponding downstream and upstream regions of various DNA modification fragments CpxRD51A, CpxRM199A, CpxRSD, CpxRN–3×FLAG, and CpxRD51A/M199A (require additional midstream), were amplified by PCR using the genomic DNA of WT strain JS. 3 × FLAG, tagging the N-terminal of CpxR, was amplified by PCR using a pairs of complementary oligonucleotides. UP, upstream region of the target gene to be modified. DW, downstream region of the target gene to be modified. MD, midstream region of the target gene to be modified. In seamless assembly, successful assembly of corresponding PCR products were confirmed by PCR with primers CpxR-UF/DR. In PCR-2, various recombinogenic DNA fragments were amplified by PCR with primers CpxR∗-F/R. Molecular size markers (DL2000 DNA marker, Takara) are indicated. [file Image_4.TIF]

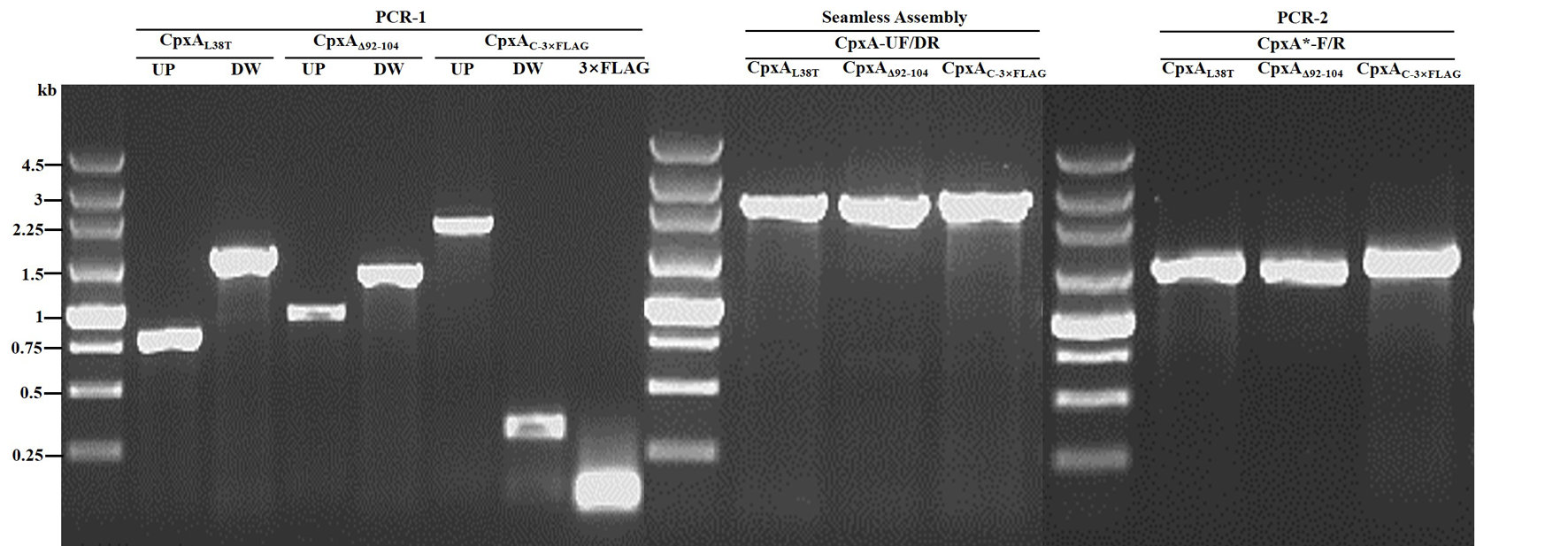

Supplement: FIGURE S5 — Preparation of various recombinogenic DNA of CpxA. In PCR-1, the corresponding downstream and upstream regions of various DNA modification fragments CpxAL38F, CpxAΔ 92–104 and CpxAC–3×FLAG, were amplified by PCR using the genomic DNA of WT strain JS. 3 × FLAG, tagging the C-terminal of CpxA, was amplified by PCR using a pair of complementary oligonucleotides. UP, upstream region of the target gene to be modified. DW, downstream region of the target gene to be modified. In seamless assembly, successful assembly of corresponding PCR products were confirmed by PCR with primers CpxA-UF/DR. In PCR-2, various recombinogenic DNA fragments were amplified by PCR with primers CpxA∗-F/R. Molecular size markers (250 bp DNA ladder marker, Takara) are indicated. [file Image_5.TIF]

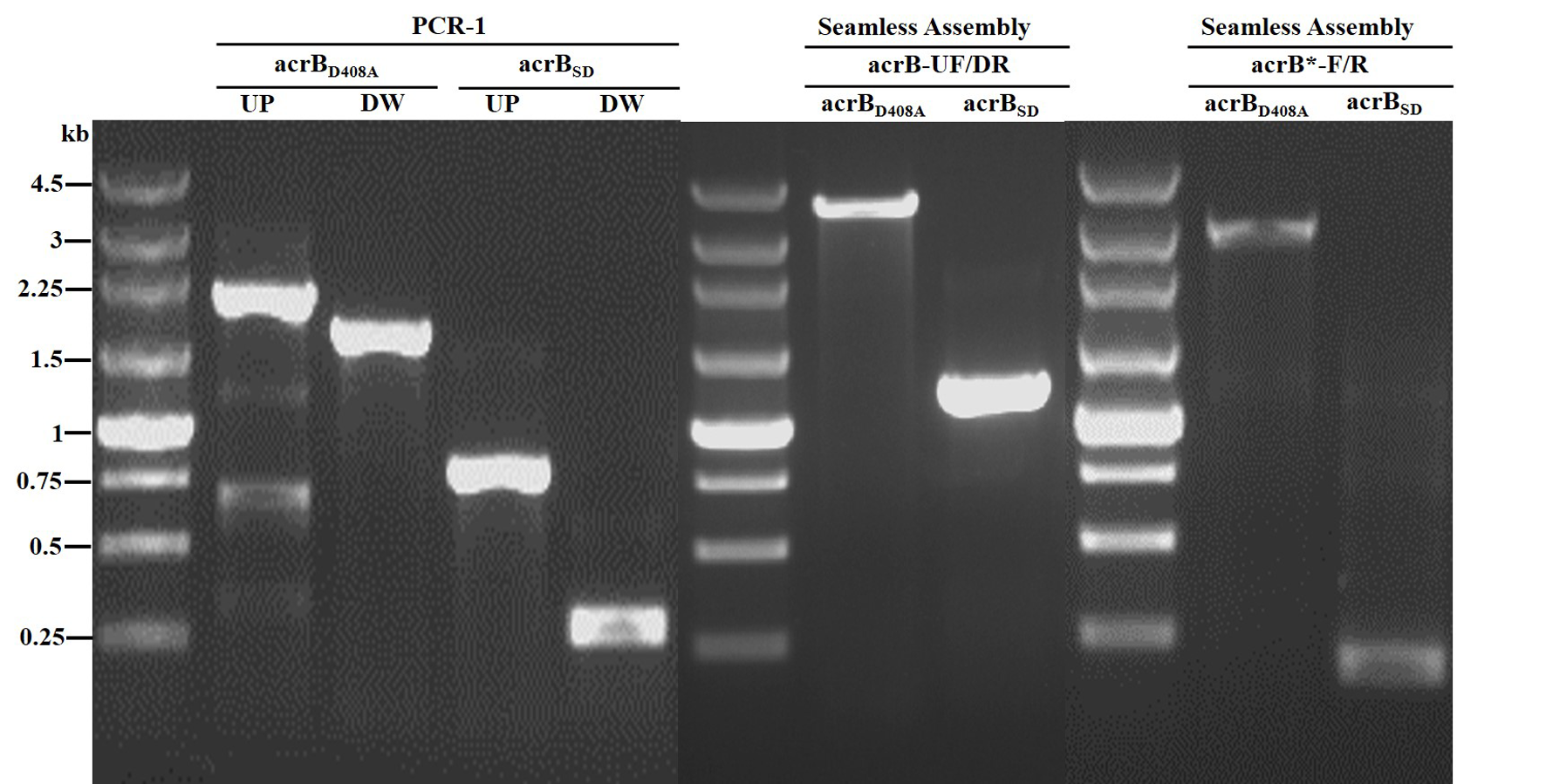

Supplement: FIGURE S6 — Preparation of various recombinogenic DNA of acrB. In PCR-1, the corresponding downstream and upstream regions of various DNA modification fragments, acrBD408A and acrBSD were amplified by PCR using the genomic DNA of WT strain JS. UP, upstream region of the target gene to be modified. DW, downstream region of the target gene to be modified. In seamless assembly, successful assembly of corresponding PCR products were confirmed by PCR with primers acrB-UF/DR. In PCR-2, various recombinogenic DNA fragments were amplified by PCR with primers acrB∗-F/R. Molecular size markers (DL2000 DNA marker, Takara) are indicated. [file Image_6.TIF]
