# Supplementary material for: Development of a Method for Simultaneous Generation of Multiple Genetic Modification in Salmonella enterica Serovar Typhimurium
Source: Front Genet. 2020 Sep 24;11:563491. doi: 10.3389/fgene.2020.563491 (PMC7544003; doi:10.3389/fgene.2020.563491)
Supplement: TABLE S1 — Primers used in this study. [file Data_Sheet_2.doc]

**Table. S1** Primers used in this study

| Primer | Sequence(5’-3’) | Description |
| --- | --- | --- |
| Construction of plasmid psacBKan |  |  |
| Kan-*Bam*H I-F | CGC*GGATCC*AAGAGTTGGTAGCTCTTGATCC | *Kan* resistance cassette |
| Kan-*Eco*R I-R | CCG*GAATTC*ATATGAATATCCTCCTTAGGGAACAACACTCAACCCTATCT |  |
| sacB-*Sph* I-F | ACAT*GCATGC*GTGTAGGCTGGAGCTGCTTCCCATGCAACAGAAACTATAA | *sacB* counterselection marker |
| sacB-*Bam*H I-R | CGC*GGATCC*AAGGTTAGGAATACGGTTAG |  |
| Mutations of *CpxR* |  |  |
| CpxR-F | GGATGGTGTCTATGGCAAGGAA | Identification of *CpxR* mutants |
| CpxR-R | ACAGAAGCGGGCGGTCAA |  |
| CpxR-H1P1 | GGATTAGCGACGCCTGATGACGTAATTTCTGCCTCGGAGGTACGTAAACAGTGTAGGCTGGAGCTGCTTC | *sacBKan* insertion fragment used for *CpxR* mutations |
| CpxR-H2P2 | CGCCAGCGTCAACCAGAAGATGGCGAAGATGCGCGCGGTTAAACTTCCTACATATGAATATCCTCCTTAG |  |
| CpxR*-F | CAACGTAAAGTCATGGATTAGCGA | Substitution modification fragment of *CpxR* |
| CpxR*-R | CATCAGGACCAGCATTAGCACC |  |
| CpxR-UF | ATTCGAGCTCGGTACCCGGGCAGCGTTGAGGCCATAACAG | Integration of modification fragment into the vector |
| CpxR-DR | CCTGCAGGTCGACTCTAGAGTCAGGTCGTTGGGCGGATC |  |
| CpxR-D51A-UR | CATCATGACGGCAAGCAAAAGTAAATCGA | D51A mutation of CpxR |
| CpxR-D51A-DF | CTTTTGCTTGCCGTCATGATGCCGAAGA |  |
| Cpxr-M199A-UR | TAGAAATATGCGCATCAATGGCGCGATCGAACG | M199A mutation of CpxR |
| Cpxr-M199A-DF | CGCCATTGATGCGCATATTTCTAACCTGCGCCG |  |
| CpxR-SD-UR | GTTCCGGCAGCGAGGAGCTCTTTTAACAGGGA | Seamless deletion of *CpxR* |
| CpxR-SD-DF | GAGCTCCTCGCTGCCGGAACGCAAAGACGG |  |
| CpxR-N3F-UR | CCGTCATGGTCTTTGTAGTCCATTGTTTACGTACCTCCGAGG | N-terminal 3×FLGA tagging of CpxR |
| CpxR-N3F-DF | ACAAGGATGACGATGACAAGAATAAAATCCTGTTAGTTGATGATGACC |  |
| Mutations of *CpxA* |  |  |
| CpxA-F | TTCGGGCTGCAAACATGC | Identification of *CpxA* mutants |
| CpxA-R | GGCTGGCTGTATCGGGTCA |  |
| CpxA-H1P1 | GATGGTTTCCGCTTCATGATAGGAAGTTTAACCGCGCGCATCTTCGTGTAGGCTGGAGCTGCTTC | *sacBKan* insertion fragment used for *CpxA* mutations |
| CpxA-H2P2 | TCGGCCTGCATTCGCAGGCCGATGGTTTTTAGGTTCGCTTGTACACATATGAATATCCTCCTTAG |  |
| CpxA*-F | GGTTTAAAACATTGCGTGGTCG | Modification substitution fragment of *CpxR* |
| CpxA*-R | GTGCCGGAAATCTCCCGGTA |  |
| CpxA-UF | ATTCGAGCTCGGTACCCGGGCGAAATGGAAGGTTTTAATGTC | Integration of modification fragment into the vector |
| CpxA-DR | CCTGCAGGTCGACTCTAGAGCGAGTTTGATGCGGTGCAG |  |
| CpxA-L38F-UR | CGCTGTCCAGAAACTCGGTCATCTGGCG | L38F mutation of CpxA |
| CpxA-L38F-DF | GATGACCGAGTTTCTGGACAGCGAACAG |  |
| CpxA-92-104-UR | AGTTACGAATTTCAGAGGTCACCAGTAATA | Deletion of 92-104 amino acid of CpxA |
| CpxA-92-104-DF | GACCTCTGAAATTCGTAACTTCATTGGTCA |  |
| CpxA-C3F-UR | CCGTCATGGTCTTTGTAGTCGGTTCGCTTGTACAGCGGTAG | C-terminal 3×FLGA tagging of CpxA |
| CpxA-C3F-DF | ACAAGGATGACGATGACAAGTAAAAACCATCGGCCTGCGAATGC |  |
| Mutations of *acrB* |  |  |
| acrB-F | CGTACCGTTGCTTACCGTATCGC | Identification of *acrB* mutants |
| acrB-R | GATGTTCTGTCGAATGACTATGC |  |
| acrB-H1P1 | TGATCAACCTGCTCAGCCCAGGTCTTAACTTAAACAGGAGCCGTTAAGACGTGTAGGCTGGAGCTGCTTC | *sacBKan* insertion fragment used for *acrB* mutations |
| acrB-H2P2 | TCATACAATGCCGCCAGACACAGGAAGACGACGATCAGCGATATAGCATACATATGAATATCCTCCTTAG |  |
| acrB*-F | GGAAATTACCGCGGATAACAAAC | Modification substitution fragment of *acrB* |
| acrB*-R | CAAGCGGAACAACCAGCATTA |  |
| acrB-UF | GAATTCGAGCTCGGTACCCGGGCGCACAACAAGCGACTGCC | Integration of modification fragment into the vector |
| acrB-DR | CCTGCAGGTCGACTCTAGAGGTCGCTTCTACCAGACCTTTCC |  |
| acrB-D408A-UR | CCACGATGGCGGCATCCACCAGCAAGCC | D408A mutation of acrB |
| acrB-D408A-DF | GCTGGTGGATGCCGCCATCGTGGTGGTC |  |
| acrB-SD-UR | ATATAGCATAGTCTTAACGGCTCCTGTTTAAG | Seamless deletion of *acrB* |
| acrB-SD-DF | CCGTTAAGACTATGCTATATCGCTGATCGTC |  |
| 3×FLAG-F | GACTACAAAGACCATGACGGTGATTATAAAGATCATGACATCGACTAC | 3×FLAG fragment |
| 3×FLAG-R | CTTGTCATCGTCATCCTTGTAGTCGATGTCATGATCTTTATAATCACC |  |
| Control primers |  |  |
| sacBKan-K1 | GCCTGGACGTTTGGGACA | Check *sacBKan* insertion |
| sacBKan-K2 | CGGATAAAATGCTTGATGGTCG |  |

Italic letters indicate the respective restriction enzyme site in the primer. The overlapping sequence is underlined.
