# Supplementary material for: Development of a Method for Simultaneous Generation of Multiple Genetic Modification in Salmonella enterica Serovar Typhimurium
Source: Front Genet. 2020 Sep 24;11:563491. doi: 10.3389/fgene.2020.563491 (PMC7544003; doi:10.3389/fgene.2020.563491)
Supplement: FILE S1 — Preparation of various recombinogenic DNA fragments. [file Data_Sheet_1.docx]

**Supplementary Methods**

**Supplemental material File S1. Preparation of various recombinogenic DNA fragments**

**1 Preparation of** **various** **recombinogenic DNA fragments for *CpxR***

(i) Recombinogenic DNA fragment *CpxR_D51A_*

In PCR-1, a 0.35-kb upstream fragment and a 0.75-kb downstream fragment of the *CpxR* gene were amplified from *S. enterica* serovar Typhimurium CVCC541 genomic DNA using the primers pairs CpxR-UF/CpxR-D51A-UR and CpxR-D51A-DF/CpxR-DR. In SA (Seamless Assembly), 5 μl mix containing both gel-purified PCR products and *Bam*HI-digested vector at 1:1:1 molar ratio and 5 μl assembly mix were then mixed in a 10 μl system. Gently mix and incubate at 50℃ for 15 min and then place it on ice for a few seconds. A 1.08-kb fragment was amplified from the positive [chimeric](javascript:;) [plasmid](javascript:;) pCpxR_D51A_, using the primer pairs CpxR-UF/CpxR-DR. Finally, in PCR-2, a 0.84-kb recombinogenic DNA fragment was amplified from the [plasmid](javascript:;) pCpxR_D51A_ with completely correct sequence, using primer pair CpxR*-F/CpxR*-R. All the PCR amplification were in 50 μl reaction system. Reaction conditions as follows: 30 cycles of 98℃ for 10s, 56℃ for 5s, and 72℃ for 30s, with a final elongation at 72℃ for 5 min.

(ii) Recombinogenic DNA fragment *CpxR_M199A_*

In PCR-1, a 0.80-kb upstream fragment and a 0.31-kb downstream fragment of the *CpxR* gene were amplified using the primers pairs CpxR-UF/CpxR-M199A-UR and CpxR-M199A-DF/CpxR-DR. In SA and PCR-2, the processes, the size of the fragments and the primers were the same as (i).

(iii) Recombinogenic DNA fragment *CpxR_D51A/M199A_*

In PCR-1, the upstream and downstream fragments were the 0.35-kb upstream fragment of *CpxR_D51A_* and the 0.31-kb downstream fragment of *CpxR_M199A_*. A 0.4-kb midstream fragment of the *CpxR* gene was amplified using the primer pair CpxR-D51A-DF/CpxR-M199A-UR. In SA, 5 μl mix containing both gel-purified PCR products and *Bam*HI-digested vector at 2:2:2:3 molar ratio and 5 μl assembly mix were then mixed in a 10 μl system. The followed processes, the size of the fragments and the primers were the same as (i).

(iv) Recombinogenic DNA fragment *CpxR_SD_*

In PCR-1, a 0.26-kb upstream fragment and a 0.28-kb downstream fragment of the *CpxR* gene were amplified using the primers pairs CpxR-UF/CpxR-SD-UR and CpxR-SD-DF/CpxR-DR. The followed processes and primers pairs were the same as (i). In SA, a 0.54 kb fragment was amplified from the positive [chimeric](javascript:;) [plasmid](javascript:;) pCpxR_SD_, using the primer pairs CpxR-UF/CpxR-DR. In PCR-2, a 0.28-kb recombinogenic DNA fragment was amplified from the [plasmid](javascript:;) pCpxR_SD_ with completely correct sequence, using primer pair CpxR*-F/CpxR*-R.

(v) Recombinogenic DNA fragment *CpxR_N-3×FLAG_*

In PCR-1, a 0.19-kb upstream fragment and a 0.89-kb downstream fragment of the *CpxR* gene were amplified DNA using the primers pairs CpxR-UF/CpxR-N3F-UR and CpxR-N3F-DF/CpxR-DR. A 0.07-kb midstream 3×FLAG fragment was amplified using two complementary primer pair 3×FLAG-F/3×FLAG-R. The followed processes and primer pairs were the same as (iii). In SA, a 0.11-kb fragment was amplified from the positive [chimeric](javascript:;) [plasmid](javascript:;) pCpxR_N-3×FLAG_, using the primer pairs CpxR-UF/CpxR-DR. In PCR-2, a 0.90-kb recombinogenic DNA fragment was amplified from the [plasmid](javascript:;) pCpxR_N-3×FLAG_ with completely correct sequence, using primer pair CpxR*-F/CpxR*-R.

**2 Preparation of various recombinogenic DNA fragments for *CpxA***

(i) Recombinogenic DNA fragment *CpxA_L38F_*

In PCR-1, a 0.76-kb upstream fragment and a 1.5-kb downstream fragment of the *CpxA* gene were amplified from *S. enterica* serovar Typhimurium CVCC541 genomic DNA using the primers pairs CpxA-UF/CpxA-L38A-UR and CpxA-L38A-DF/CpxA-DR. In SA, 5 μl mix containing both gel-purified PCR products and *Bam*HI-digested vector at 1:1:1 molar ratio and 5 μl assembly mix were then mixed in a 10 μl system. Gently mix and incubate at 50℃ for 15 min and then place it on ice for a few seconds. A 2.26-kb fragment was amplified from the positive [chimeric](javascript:;) [plasmid](javascript:;) pCpxA_L38F_, using the primer pairs CpxA-UF/CpxA-DR. Finally, in PCR-2, a 1.49-kb recombinogenic DNA fragment was amplified from the [plasmid](javascript:;) pCpxA_L38F_ with completely correct sequence, using primer pair CpxA*-F/CpxA*-R.

(ii) Recombinogenic DNA fragment *CpxA_Δ92-104_*

In PCR-1, a 0.92-kb upstream fragment and a 1.32-kb downstream fragment of the *CpxA* gene were amplified using the primers pairs CpxA-UF/CpxA-92-104-UR and CpxA-92-104-DF/CpxA-DR. The followed processes and primers pairs were the same as (i). In SA, a 2.2-kb fragment was amplified from the positive [chimeric](javascript:;) [plasmid](javascript:;) pCpxA_Δ92-104_, using the primer pairs CpxA-UF/CpxA-DR. Finally, in PCR-2, a 1.45-kb recombinogenic DNA fragment was amplified from the [plasmid](javascript:;) pCpxA_Δ92-104_ with completely correct sequence, using primer pair CpxA*-F/CpxA*-R.

(iii) Recombinogenic DNA fragment *CpxA_C-3×FLAG_*

In PCR-1, a 2.03 kb upstream fragment and a 0.28 kb downstream fragment of the *CpxA* gene were amplified DNA using the primers pairs CpxA-UF/CpxA-C3F-UR and CpxA-C3F-DF/CpxA-DR. A 0.07 kb midstream 3×FLAG fragment was amplified using two complementary primer pair 3×FLAG-F/3×FLAG-R. The followed processes and primer pairs were the same as (i). In SA, a 2.32-kb fragment was amplified from the positive [chimeric](javascript:;) [plasmid](javascript:;) pCpxA_C-3×FLAG_, using the primer pairs CpxA-UF/CpxA-DR. In PCR-2, a 1.56-kb recombinogenic DNA fragment was amplified from the [plasmid](javascript:;) pCpxA_C-3×FLAG_ with completely correct sequence, using primer pair CpxA*-F/CpxA*-R.

**3 Preparation of various recombinogenic DNA fragments for *acrB***

(i) Recombinogenic DNA fragment *acrB_D408A_*

In PCR-1, a 2.01-kb upstream fragment and a 1.69-kb downstream fragment of the *acrB* gene were amplified from *S. enterica* serovar Typhimurium CVCC541 genomic DNA using the primers pairs acrB-UF/acrB-D408A-UR and acrB-D408A-DF/acrB-DR. In SA, 5 μl mix containing both gel-purified PCR products and *Bam*HI-digested vector at 1:1:1 molar ratio and 5 μl assembly mix were then mixed in a 10 μl system. Gently mix and incubate at 50℃ for 15 min and then place it on ice for a few seconds. A 3.69-kb fragment was amplified from the positive [chimeric](javascript:;) [plasmid](javascript:;) pacrB_D408A_, using the primer pairs acrB-UF/acrB-DR. Finally, in PCR-2, a 2.81-kb recombinogenic DNA fragment was amplified from the [plasmid](javascript:;) pacrB_D408A_ with completely correct sequence, using primer pair acrB*-F/acrB*-R.

(ii) Recombinogenic DNA fragment *acrB_SD_*

In PCR-1, a 0.78-kb upstream fragment and a 0.27-kb downstream fragment of the *acrB* gene were amplified using the primers pairs acrB-UF/acrB-SD-UR and acrB-SD-DF/acrB-DR. In SA, A 1.03-kb fragment was amplified from the positive [chimeric](javascript:;) [plasmid](javascript:;) pacrB_SD_ using the primer pairs acrB-UF/acrB-DR. Finally, in PCR-2, a 0.18-kb recombinogenic DNA fragment was amplified from the [plasmid](javascript:;) pacrB_SD_ with completely correct sequence, using primer pair acrB*-F/acrB*-R.
